# Treatment of AL amyloidosis in the era of novel immune and cellular therapies

**DOI:** 10.3389/fonc.2024.1425521

**Published:** 2024-06-28

**Authors:** Caitlin Sarubbi, Hesham Abowali, Cindy Varga, Heather Landau

**Affiliations:** ^1^ Department of Medicine, Montefiore Medical Center/Albert Einstein College of Medicine, Bronx, NY, United States; ^2^ Department of Hematology/Oncology, Brookdale University Medical Center, Brooklyn, NY, United States; ^3^ Department of Hematology, Levine Cancer Institute Atrium Health, Charlotte, NC, United States; ^4^ Department of Medicine, Memorial Sloan Kettering Cancer Center, New York, NY, United States

**Keywords:** AL amyloidosis, bispecific antibodies, CAR T cell therapy, stem cell transplant, daratumumab

## Abstract

Light chain (AL) amyloidosis is a plasma cell disorder distinguished from multiple myeloma (MM) by the degree of organ involvement due to tissue deposition of misfolded proteins. Treatments for AL amyloidosis have largely been borrowed from those developed for patients with MM. High-dose chemotherapy followed by autologous stem cell transplant (ASCT) has historically been associated with the best outcomes. The recent incorporation of daratumumab into up front therapy represents a significant advance and has changed the treatment paradigm, calling into question the role of ASCT. The development of very active novel immune and cellular therapies, specifically B cell maturation antigen (BCMA)-directed therapies, has similarly been transformative for patients with MM and is now being studied in patients with AL amyloidosis. These include chimeric antigen receptor (CAR) T cells, bispecific antibodies, and antibody drug conjugates. Although limited, preliminary data in patients with relapsed and refractory AL amyloidosis are showing promising results, and it is expected that the treatment landscape for AL amyloidosis will continue to evolve. Particular attention to safety, potential for organ recovery, and quality of life will be important when evaluating new treatments and/or treatment paradigms.

## Introduction

Light chain (AL) amyloidosis is a plasma cell disease in which light chains that are produced misfold and deposit in various tissues and organs throughout the body. Although the degree of plasma cell burden is often minimal compared to multiple myeloma, the considerable morbidity and mortality associated with AL amyloidosis result from organ dysfunction (1, 2). While light chain proteins can deposit in any organ, the most affected organs are the heart (80% of patients), followed by the kidneys (60%–70% of patients) (3). The amyloid proteins that are circulating and not yet deposited in the organs are also toxic. In the case of AL amyloidosis, serum free light chain levels are closely correlated with the degree of disease (2, 3).

Most treatment options for AL amyloidosis are aimed at decreasing the serum levels of light chains with the use of anti-plasma cell agents. Survival is predicted by the organ affected as well as the depth and speed of hematologic response, which are necessary for organ function to be restored (4). Autologous stem cell transplant (ASCT) has been associated with rapid and durable suppression of the plasma cell clone and therefore, has been a commonly used therapeutic option in AL amyloidosis. Many aspects of this treatment paradigm, such as patient selection, melphalan dosing, and peri-transplant supportive care, have been adapted over time to reduce toxicity and improve outcomes. However, with the availability of novel plasma cell-directed therapies such as anti-CD38 antibodies, there is ongoing research looking at the optimal use and timing of ASCT as well as alternative treatment options. This review will examine the role of ASCT and anti-BCMA agents, including T cell redirecting therapies, in the treatment of patients with AL amyloidosis.

## Autologous stem cell transplant

High-dose melphalan conditioned ASCT has been a mainstay of treatment for patients with AL amyloidosis for nearly 30 years, modeled after its use in multiple myeloma. In 1996 a pilot study was performed in which five patients with amyloid underwent ASCT, of which 60% achieved a hematologic complete response (CR), and all five patients had improvement in their involved organ function (5). These results were expanded upon in 2004 when a group of 312 patients underwent ASCT, resulting in a 40% CR rate and 66% organ response (6). Recent data indicate that approximately two-thirds of patients who undergo ASCT are alive 10 years following transplant, and among those who achieve a CR, almost 50% are event-free 15 years later (7).

While high-dose melphalan was the first treatment to result in meaningful hematologic responses, transplant related mortality (TRM) was higher in patients with AL amyloidosis than had been observed in MM patients (8). Patients with cardiac disease, renal impairment, multiorgan involvement, and/or advanced age were at particular risk for transplant related morbidity and mortality. To reduce toxicity, the melphalan dose was attenuated with melphalan 200 mg/m^2^ or 100–140 mg/m^2^ given to patients without risk factors versus those with one or more risk factors, respectively. Several studies have reported higher CR rates and better outcomes with melphalan 200 mg/m^2^ when compared to reduced doses of melphalan (9, 10). Lower response rates in patients receiving attenuated doses of melphalan have led to the notion that high-dose therapy should be reserved for patients who can receive the full dose (11). However, several publications show the benefit of modified melphalan dosing in patients with AL amyloidosis (12–14). Among a series of 143 patients who received risk-adapted melphalan dosing, only 34% of patients received melphalan 200 mg/m^2^. The OS of all patients surpassed 10 years (15). The absence of early death in patients who received a full dose of melphalan suggests that host factors necessitating dose reductions were primarily responsible for TRM rather than the dose of melphalan itself. The 100-day TRM rate was only 5% in that series, compared to up to 30% when dose reduction was not considered, indicating the safety of this approach (15, 16).

The advent of propylene glycol-free (PG-free) melphalan (Evomela™), resulting in improved stability compared to standard melphalan, has allowed for pharmacokinetically (Pk)-directed dosing in patients with plasma cell disorders (17). Safety and efficacy outcomes in patients with AL amyloidosis conditioned with PG-free melphalan are similar to those using the standard formulation (18). Pk-directed dosing has the potential to optimize and individualize melphalan conditioning, which may be particularly important in patients with AL amyloidosis but will require further study in randomized trials (19).

In addition to attenuated melphalan dosing, stricter selection criteria are associated with reduced toxicity of ASCT for patients with AL amyloidosis and an improved safety profile. A working group designated by the International Society of Amyloidosis (ISA) and the European Hematology Association (EHA) recently put forth eligibility guidelines (see **Table 1**). Definite exclusions include decompensated heart failure, pleural effusions, medically refractory ventricular arrhythmias and/or orthostatic hypotension, and extensive gastrointestinal involvement or Factor X deficiencies that would pose an excess risk of bleeding. While compromised renal function is not a definite exclusion, melphalan should be adjusted based on a reduced estimated glomerular filtration rate (eGFR), and the risk of worsening renal function in the peri-ASCT period must be contemplated. Given these considerations, only 20%–30% of newly diagnosed patients with AL amyloidosis are eligible for transplantation, but that percentage is likely dynamic based on recent advances in the field (8).

**Table 1:** Broad eligibility criteria for ASCT for AL amyloidosis (8).

**Diagnostic criteria**	Confirmed tissue amyloid diagnosis and typing
	Evidence of plasma cell disorder
	At least 1 organ involved
**Performance criteria**	ECOG performance status < 2 (unless limited by peripheral neuropathy)
**Age**	18–70
**Cardiac and pulmonary criteria**	Left ventricular ejection fraction > 40%
	NYHA class < III
	DLCO > 50%
	Supine systolic blood pressure > 90 mmHg
	NTpro BNP < 5,000 pg/mL
	Troponin I < 0.1 ng/mL and troponin *T* < 60 ng/mL and hs-troponin *T* < 75 ng/mL
	Oxygen saturation > 95% on room air
**Hepatic criteria**	Direct bilirubin < 2 mg/dL
**Renal criteria**	eGFR > 30 mL/min/m^2^

Melphalan 140 mg/m^2^ for estimated glomerular filtration rate (eGFR) < 50 mL/min/m^2^, age > 70, and/or Mayo cardiac stage > II.

Given the multiorgan involvement and complex pathophysiology associated with AL amyloidosis, a multidisciplinary care team is ideal to properly support patients, and thus, the experience of the transplant center matters. Institutes that perform less than 4 transplants for AL amyloid patients per year have more than double the TRM at 100 days post-transplant, compared to experienced centers that perform more (20).

The preponderance of evidence suggests that the incorporation of a fixed and limited number of cycles of bortezomib-based induction prior to ASCT has led to more frequent hematologic responses and superior outcomes following ASCT compared to ASCT alone (21–24). The risk of toxicity from induction therapy should be individually weighed against the benefit so as not to compromise a patient’s eligibility for ASCT.

In 2021, the historic ANDROMEDA study demonstrated that the addition of daratumumab to bortezomib, cyclophosphamide, and dexamethasone (VCd) as induction therapy resulted in deeper hematologic responses, increased organ responses, and better outcomes compared to VCd alone (25). Remarkably, hematologic CRs were achieved in over 50% of patients who received Dara-VCd, and these responses occurred quickly (the median time to CR was 60 days). Based on these data, Dara-VCd has become the first (and only) FDA-approved induction regimen for the treatment of AL amyloidosis and is now widely accepted as a standard of care. With such effective induction therapy, the role of ASCT in the frontline setting is uncertain. Prompt hematologic disease control with Dara-VCd may improve a patient’s condition, potentially increasing the number of patients who are transplant-eligible. Alternatively, if a CR is achieved with two to four cycles of induction, some physicians and patients may opt to defer ASCT (8). Although the results of the ANDROMEDA study are promising, it should be recognized that there are no data on the long-term outcomes of daratumumab-based induction therapies, and whether ASCT following Dara-VCd induction will improve the depth and duration of response has not been tested. The recently initiated SWOG trial (S2213) (NCT06022939), a phase III randomized study of Dara-VCd induction followed by ASCT versus Dara-VCd consolidation in patients with newly diagnosed AL amyloidosis, was designed to answer this question, but results are not expected for several years.

The results of the ANDROMEDA study have positively impacted the treatment of patients with AL amyloidosis. With the rapid development of alternate immune-based and cellular therapies for plasma cell disease, the treatment landscape will continue to evolve. Particular attention to safety, potential for organ recovery, and quality of life will be important when evaluating new treatments and/or treatment paradigms.

## Novel immune and cellular therapies

### CAR T cell therapy

While daratumumab-based initial therapy represents a significant advance in the treatment of AL amyloidosis, nearly half of patients do not achieve CR (47%), and organ responses are not guaranteed (25). Hematologic response rates in patients who fail to respond or relapse following daratumumab-based therapies are even lower (26). High-dose melphalan and ASCT are options for salvage therapy, but only for the percentage of patients who are transplant-eligible. Thus, second-line therapy for patients with AL amyloidosis is a critical unmet medical need.

Chimeric antigen receptor (CAR) T cell therapy is a novel therapeutic strategy utilizing T cells that are genetically engineered to target and destroy cells or tissues expressing a particular antigen. The unprecedented efficacy of B cell maturation antigen (BCMA)-directed CAR T cells in heavily pre-treated MM has led to the FDA-approval of two products, idecabtagene vicleucel and citacabtagene autoleucel, for this indication. While overall and complete hematologic response rates were notably high, ranging from 73% to 97% and 33% to 67%, respectively, these products have unique toxicities, including cytokine release syndrome (CRS) and immune effector cell associated neurotoxicity syndrome (ICANS), that require specialized management (27–29).

Similar to MM, BCMA is highly expressed on amyloidogenic plasma cells and also retained at relapse (30). As the risk of CRS and ICANS was expected to pose a significant risk to patients with AL amyloidosis, these patients have been notably excluded from initial studies. However, emerging data suggest that CAR T cells can be safely administered and have promising activity in this patient population.

The first case report from Spain described a patient with MM who developed AL amyloidosis with renal involvement in the context of relapsing disease (31). The patient received an academic BCMA CAR T cell product under compassionate use (NCT04309981) and developed grade 1 CRS that resolved within 48 h without treatment without signs or symptoms of ICANS. By D+28, the patient achieved a hematologic very good partial response (VGPR) (normal sFLCs and small residual M-spike) and had no detectable disease (minimal residual disease (MRD) negative) in the bone marrow. The M-spike resolved by D+90, and by 6 months following CAR T cell infusion, she achieved a renal response. It is now almost 4 years (46 months) following her CAR T cell infusion, and this patient remains in CR with minimal proteinuria (< 200 mg).

Two other patients with relapsed refractory MM and coexisting cardiac and renal AL amyloidosis were reported by the Mayo Jacksonville group (32). Given the initial concern for adverse effects, patients were given CRS prophylaxis according to the ZUMA-1 trial in the form of dexamethasone (33). The first patient was penta-refractory after eight lines of therapy and received commercially available idecabtagene vicleucel. During the hospitalization, the patient developed anemia and neutropenia but no evidence of CRS, ICANS, or organ dysfunction. By D+30, the patient achieved a hematologic VGPR with no MRD in the bone marrow. She had a cardiac response (> 30% reduction in NT-ProBNP) and stable renal disease following CAR T cells (32). The second patient received ciltacatagene autoleucel after four prior lines of treatment. Despite pre-treatment with dexamethasone, the patient developed grade 3 CRS and required ICU admission. With the administration of tocilizumab and IV steroids, CRS resolved within 24 h. The patient developed pancytopenia but no ICANS or cardiac dysfunction. At D+30, he achieved MRD negative disease in the bone marrow and a serologic CR. At 9 months, following CAR T cells, the patient achieved a cardiac response (32).

A phase Ia/b study (NCT04720313) of a novel anti-BCMA CAR T cell therapy developed at Hadassah Medical Center was the first prospective trial to include patients with AL amyloidosis and reported on the safety and feasibility of this approach (34). These data were updated at the American Society of Hematology meeting (December 2023) and included eleven patients (median age: 64, range: 55–82), with either cardiac (*N*= 9; Mayo stage 2 (*N*= 4), 3a (*N*= 3), or 3b (*N*= 1)) or renal (*N*= 7) involvement. The median time from diagnosis was 4.5 years (range: 0.8–11 years), median lines of prior therapy were six (range: 3–10); and all were refractory to their last line of therapy. Patients received doses of 150 ×10^6^(*N*= 1), 450 × 10^6^(*N*= 2), and 800 × 10^6^(*N*= 8).

CRS was seen in nine of eleven patients (grade 1 (*N*= 3), grade 2 (*N*= 4), grade 3 (*N*= 2)) and occurred within 1–3 days (median: 1.5) and persisted 1–4 days (median 1 day). Tociluzimab was used in seven patients and dexamethasone in two patients. No grade 4 CRS or ICANS were seen. Ten patients were evaluable for hematologic response, and 100% of patients responded including nine of ten with VGPR (*N*= 2) or CR (*N*= 7), and 67% (six of nine) had no evidence of MRD in the bone marrow by D+30. In total, 60% of patients achieved organ responses, including four cardiac and two renal responses. Follow up ranged from 0.5 to 25.2 months, and five patients remained alive with the longest response duration and survival of 25.2 months. Six patients died during the follow up period (median OS: 6.9 months, range: 5.2–12.2), five of six due to cardiac disease, three in the setting of hematologic progression, and one due to COVID while still in a CR (35).

These data provide evidence that this novel therapy is safe for patients with AL amyloidosis, including those with advanced cardiac disease, without early mortality. Hematologic responses are seen in most patients and can be deep and durable. Deaths were overwhelmingly cardiac-related, arguing for earlier use in the course of the disease.

### Bispecific antibodies

Bispecific antibodies targeting CD3 expressed on the surface of T cells and BCMA expressed on MM cells represent another effective T cell redirecting strategy that has demonstrated promising results in MM, leading to the FDA approval of two commercially available products, teclistimab and elranatamab (36, 37). In heavily pre-treated patients, both BCMA-directed bispecific antibodies showed rapid and deep responses with ORR ~ 61%–63%, including 35%–39% CR rates, with a median time to first response of 1.2 months. While CRS occurred in 56%–72% of patients, these events occurred early and were almost exclusively grades 1–2.

Patients with AL amyloidosis were excluded from the pivotal trials that led to the regulatory approval of these drugs in MM. However, once commercially available, there have been several reports demonstrating the safety and efficacy of teclistimab in patients with AL amyloidosis (38–40). A 76-year-old woman with Mayo cardiac stage IIIb (by modified European criteria), renal stage II, and soft tissue involvement with AL amyloidosis was treated with teclistimab after receiving six prior lines of therapy. She had no CRS, neurotoxicity, or other adverse effects and, after two cycles, achieved a hematologic CR and a cardiac biomarker response (38).

In another series, seven patients with AL amyloidosis from two US medical centers with cardiac (*N*= 5, Mayo cardiac stage II (2), IIIa (1), or IIIb (2)) or renal (*N*= 5) involvement, having received a median of six prior lines of therapy (range: 2–7), including four with prior BCMA-targeted therapy, were reported by Chakraborty et al. (39). All seven patients achieved at least a VGPR with a median time to response of only 0.6 months, including six of seven with a stringent dFLC response (defined by dFLC < 1 mg/dL). Among patients evaluable for organ response, three of four achieved a cardiac response. CRS occurred in four patients, all grade 1, with only one patient requiring tocilizumab. No patients had ICANS. Two patients experienced grade 3 infections, but there were no infection-related deaths. One patient with Mayo stage IIIb cardiac disease died ~ 40 days after teclistimab due to progressive cardiac dysfunction despite achieving a hematologic VGPR (39).

Forgeard et al. identified seventeen patients with AL amyloidosis from ten university hospitals in Europe who were treated with teclistimab. In total, 94% of patients had cardiac involvement (including Mayo cardiac stages IIIa (*N*= 6) and IIIb (*N*= 4), and 59% had renal involvement. After a median of three cycles (range: 0.25–10) of teclistamab, 88% of patients achieved a VGPR or better, including 41% with CR. The median time to hematologic response was approximately 1 month, and five patients achieved organ responses, four of which were cardiac responses. CRS occurred in nine patients (53%), all grade 1. One patient with a pre-existing inflammatory syndrome developed grade 3 ICANS and discontinued treatment; one patient died from cardiac progression; and one patient died from infection (40).

The advantage of bispecific antibodies over CAR T cell therapy is that they are an “off-the-shelf” product, available for immediate administration. CAR T cell therapy is hampered by significant logistical considerations, including the requirement for apheresis, prolonged waiting periods for manufacturing, the need for bridging therapy, and the high cost of treatment. However, like CAR T cells, CRS, ICANS, and infections are potential toxicities of bispecific antibodies that will require careful attention and prompt management in patients with AL amyloidosis.

### Antibody drug conjugates

Antibody drug conjugates (ADCs) are composed of an antibody bound to a cytotoxic drug through a chemical linker. Belantamab mafadotin is an ADC with an antibody against BCMA that delivers a microtubule inhibitor, monomethyl auristatin F, to pathologic plasma cells (41).

Zhang et al. identified six patients with relapsed or refractory AL amyloidosis, with a median age of 61 years (range: 51–74), a median of six lines of prior therapy (range: 5–10), who were treated with belantamab mafadotin. Five of these patients had cardiac involvement. Overall, five patients (83%) achieved a hematologic response, including (three of six) 50% achieving a CR. Time to response was rapid (ranging from 1 week to 5 months), and four of five evaluable patients achieved a cardiac response. Keratopathy occurred in five of six (83%) patients but was all grade 1 (*N*= 2) and 2 (*N*= 3) (42).

A larger retrospective series that included 31 patients with relapsed and refractory AL amyloidosis treated in the UK with belantamab mafadotin was reported by Khwaja et al. (43). Patients had a median age of 65 years (range: 41–78), received a median of three prior lines of therapy (range: 1–6), and had renal (*N*= 23, 74%) and cardiac (*N*= 18, 58%) involvement, including Mayo stages IIIa (*N*= 5) and IIIb (*N*= 4). The ORR was 71% (22/31) and included 35% (11/31) and 23% (7/31) with CR and VGPR, respectively. The median time to hematologic response was 2 months. At 12 months following initiation of treatment, 65% of patients remained on belantamab. The most common reasons for treatment discontinuation were inadequate response or progression (*N*= 7) and physician choice (*N*= 3). Two patients died, one due to the progression of the disease and another due to an unrelated cause. The most frequent toxicity was corneal keratopathy, which was observed in (21/31) 68%. Keratopathy was mostly low grade (grades 1/2/3/4; *N*= 7/10/3/4, respectively) and improved in all patients after a delay in treatment (43).

An ongoing phase II clinical trial studying belantamab in AL amyloidosis patients is being conducted by the European Myeloma Network (EMN27; NCT04617925), and interim results were presented at the American Society of Hematology meeting in December 2023. At the time, 28 patients with a median age of 66 years (range: 46–80) and a median of four (range: 1–10) prior lines of therapy were enrolled. Cardiac and renal involvement were present in 22 (79%) and 17 (61%) of the patients, respectively. Overall hematologic response was achieved in 54% (15/28) of patients, including nine (32%) with VGPR, with a median time to first response of 15 days (range: 7–148). Ocular adverse events were common, observed in 96% of patients, including grade 3/4 events in up to 40% of patients. Visual symptoms were frequent in this study despite extending the dosing from the standard every 3 weeks administration schedule to every 6 weeks from treatment initiation and dose reductions/delays (44).

**Table 2** summarizes the available literature on BCMA-targeted therapies in AL amyloidosis.

**Table 2:** BCMA-directed therapies in AL amyloidosis: literature review.

	*N*	Follow up (median; months)	Organs involved	Hematologic response	Organ response	Adverse effects of interest (CRS, ICANS or neurotoxicity, serious infection, ocular toxicity)
CAR T cell therapy						
Oliver-Caldes et al.	1	12	Renal	CR	Renal	CRS (grade 1)
Das et al.	2	9	Pt 1: cardiac and renal Pt 2: cardiac	Pt 1: VGPR^c^ Pt 2: CR^c^	Pt 1: cardiac Pt 2: cardiac	Pt 2: CRS (grade 3)
Lebel et al.^a^	11	6 (range: 0.5–25.2)	Cardiac: 9/11 Renal: 7/11	CR: 7/10^a^ VGPR: 2/10^a^	Cardiac: 4/9 Renal: 2/7	CRS: 9/11 (grades 1/2/3:3/4/2) 1 grade 5 COVID infection
Bispecific antibodies						
Chakraborty et al.	7	3.2 (range: 1.4–9)	Cardiac: 5/7 Renal: 5/7	CR: 3/7 VGPR: 4/7^d^	Cardiac: 3/4 Renal: 1/2	CRS: 4/7 (all 4 grade 1)
Forgeard et al.	17	N/A	Cardiac: 16/17 Renal: 10/17	CR: 7/17 VGPR: 8/17	Cardiac: 4/16 Renal: 1/10	CRS: 9/17 (all 9 grade 1) ICANS: 1/17 (grade 3) 1 grade 5 bacterial infection
Leung et al.	1	6	Cardiac Renal	CR	Cardiac	None
Antibody drug conjugates						
Zhang et al.	6	4.5	Cardiac: 5/6	CR: 3/6 VGPR: 2/6	Cardiac: 4/5	Keratopathy: 5/6 (grades 1/2: 2/3)
Khwaja et al.	31	12 (range: 1–29)	Cardiac: 18/31 Renal: 23/31	CR: 11/31 VGPR: 7/31	Cardiac: 1/1^g^	Keratopathy: 21/31 (grades 1/2/3/4: 7/10/3/1)
Kastritis et al.^b^	28	16	Cardiac: 22/28 Renal: 17/28 Peripheral nerve: 6/28	VGPR: 9/28 > PR: 15/28	Cardiac^e^: 3 Renal^e^: 2	Keratopathy^f^: 22/28 Grade 1/2/3/4: 10/4/4/0

a Lebel et al. phase 1a/b study; 10/11 patients are evaluable for hematologic response and six of seven patients who achieved CR had no detectable disease (MRD negative) in the bone marrow.

b Kastritis et al. phase II study.

c Both patients with hematologic VGPR and CR had no detectable disease (MRD negative) in the bone marrow.

d All patients achieved at least VGPR; CR was limited by the absence of urine protein electrophoresis/immunofixation in four patients.

e The authors report that at 3 months, three patients with cardiac and two patients with renal involvement.

f Ocular disorders including reduced visual acuity, visual impairment, and keratopathy occurred in 27/28 patients.

g Only one patient was evaluable.

## Discussion

With the advent of potent immune and cellular therapies, the treatment landscape for patients with AL amyloidosis will continue to evolve. The most mature data suggest that high-dose melphalan and ASCT can provide long-term disease control in carefully selected patients with AL amyloidosis. With improvements in supportive care, more refined eligibility criteria, and transplants at experienced centers, outcomes are ever-improving. However, only a minority of patients are eligible for ASCT in the upfront setting. It remains unclear whether achieving rapid disease control with the combination of Dara-VCd will result in comparable outcomes to ASCT in eligible patients or if consolidation with ASCT following Dara-VCd will result in additional benefits. This remains the most pressing unanswered question clinicians face in practice today. The recently initiated SWOG trial (S2213) (NCT06022939) is randomizing newly diagnosed transplant-eligible patients with AL amyloidosis to Dara-VCd with or without ASCT and will provide critically important insight on this topic. For patients who are not transplant-eligible and receive upfront Dara-VCd, the high hematologic response rate and subsequent organ improvement may increase the pool of patients who become transplant-eligible. Understanding which patients and at what level of response the risk:benefit profile favors proceeding with ASCT will continue to pose a challenge as active salvage therapies emerge.

A key advantage of CAR T cell therapy is the current “one and done” paradigm, where responding patients can experience a real treatment-free interval. Most bispecific antibodies have been studied as ongoing therapy in MM; however, in a disease like AL amyloidosis with minimal tumor burden in the bone marrow, the need for continuous therapy comes into question and should be weighed against the long-term toxicity, most notably the high risk of infections. Given the deep responses that have been reported in AL patients, perhaps a MRD-driven approach would be the most sensical: discontinuation of the drug if an MRD negative status is achieved in the bone marrow or even a de-escalation to monthly dosing once light chains have normalized. Nevertheless, aggressive supportive care to prevent infections needs to be adopted when administering T cell redirecting therapy, and this includes prophylactic antiviral therapy, medications to prevent PJP pneumonia, and IVIG replacement. While the activity of belantamab mafadotin in patients with AL amyloidosis is promising, strategies to mitigate ocular toxicity will be important to prevent decrements in patients’ quality of life and are currently being studied in MM.

Other targets that have been studied in MM, such as GPRC5D, FCHR5, and CS1/SLAM F7, may also be important in patients with AL amyloidosis, and novel therapies directed at these antigens may further expand the treatment armamentarium.

In summary, the upcoming SWOG trial (S2213) will be important to understand the role of ASCT in the setting of daratumumab-based induction and identify subgroups most likely to benefit. In the absence of a standard of care beyond first-line therapy, it is expected that CAR T cells and other immunotherapies will be studied in patients who fail to achieve an optimal hematologic or organ response to initial therapy. Patient selection, safety considerations, resistance mechanisms, organ involvement, potential for organ recovery, and quality of life implications will be critical to determining how to use and sequence novel therapies.

## Author contributions

HL: Writing – original draft, Writing – review & editing. CS: Writing – original draft, Writing – review & editing. HA: Writing – original draft, Writing – review & editing. CV: Writing – original draft, Writing – review & editing.
